# MRI Volumetric Analysis of the Hypothalamus and Limbic System across the Pediatric Age Span

**DOI:** 10.3390/children10030477

**Published:** 2023-02-27

**Authors:** Matthias W. Wagner, Patricia P. Rafful, Logi Vidarsson, Birgit B. Ertl-Wagner

**Affiliations:** 1 Division of Neuroradiology, Department of Diagnostic Imaging, The Hospital for Sick Children (SickKids), Toronto, ON M5G 1X8, Canada; 2 Neurosciences & Mental Health Research Program, SickKids Research Institute, Toronto, ON M5G 1X8, Canada; 3 Department of Medical Imaging, University of Toronto, Toronto, ON M5G 1X8, Canada; 4 Roberts Center for Pediatric Research, The Children’s Hospital of Philadelphia, Philadelphia, PA 19146, USA

**Keywords:** volumetry, MRI, brain, limbic system, children

## Abstract

Purpose: Literature is scarce regarding volumetric measures of limbic system components across the pediatric age range. The purpose of this study is to remedy this scarcity by reporting continuous volumetric measurements of limbic system components, and to provide consistent stratification data including age-related trajectories and sex-related differences in the pediatric age range in order to improve the recognition of structural variations that might reflect pathology. Methods: In this retrospective study, MRI sequences of children with normal clinical MRI examinations of the brain acquired between January 2010 and December 2019 were included. Isotropic 3D T1-weighted were processed using FreeSurfer version 7.3. Total brain volume and volumes of the limbic system including the hippocampus, parahippocampal gyrus, amygdala, hypothalamus, cingulate gyrus, entorhinal cortex, anteroventral thalamic nucleus, and whole thalamus were assessed. Parcellated output was displayed with the respective label map overlay and images were visually inspected for accuracy of regional segmentation results. Continuous data are provided as mean and standard deviation with quadratic trendlines and as mean and 95% confidence intervals. Categorical data are presented as integers and percentages (%). Results: A total of 724 children (401 female, 55.4%), with a mean age at time of MRI of 10.9 ± 4.2 years (range: 1.9–18.2 years), were included in the study. For females, the total brain volume increased from 955 ± 70 mL at the age of 2–3 years to 1140 ± 110 mL at the age of 17–18 years. Similarly, the total brain volume increased for males from 1004 ± 83 mL to 1263 ± 96 mL. The maximum volume was noted at 11–12 years for females (1188 ± 90 mL) and at 14–15 years for males (1310 ± 159 mL). Limbic system structures reached their peak volume more commonly between the 13–14 years to 17–18 years age groups. The male cingulate gyrus, entorhinal cortex, and anteroventral thalamic nucleus reached peak volume before or at 9–10 years. Conclusion: This study provides unique age- and sex-specific volumes of the components of the limbic system throughout the pediatric age range to serve as normal values in comparative studies. Quantification of volumetric abnormalities of the limbic system on brain MRI may offer insights into phenotypical variations of diseases and may help elucidate new pathological phenotypes.

## 1. Introduction

The limbic system plays an essential role in olfaction, spatial and long-term memory, learning, motivation, emotion, and in the regulation of autonomic functions including heart rate and blood pressure [1]. It is connected to and interacts with the basal ganglia, orbitofrontal cortex, and medial prefrontal cortex [1,2]. There is no unique universal agreement on the number of anatomical structures comprising the limbic system. Structures often considered part of the limbic system include the cingulate gyrus, parahippocampal gyrus, hippocampal formation (dentate gyrus, hippocampus proper, subicular complex), amygdala, septal area, and hypothalamus [1]. Torrico et al. also included the anterior thalamic nuclei, habenular commissure, entorhinal cortex, olfactory bulbs, fornix, columns of the fornix, and mammillary bodies [3]. Knowledge of the intricate structural and functional anatomy of the limbic system, its connectedness, and relationships to other brain regions is essential to better understand various disorders including schizophrenia, epilepsy, limbic encephalitis, dementia, anxiety disorders, and others [1]. Recently, Wu et al. showed that automated volumetry of T1-weighted MR images can help elucidate the neuroanatomical correlate of Prader–Willi syndrome [4]. Prader–Willi syndrome is a genetic obesity disease characterized by an insatiable appetite, hyperphagia, and morbid to life-threatening obesity [4]. In a cohort of 12 children with Prader–Willi syndrome, 18 obese children without Prader–Willi syndrome, and 18 healthy controls, Wu et al. showed that children with Prader–Willi syndrome have a significantly smaller thalamus, globus pallidus, hippocampus, amygdala, nucleus accumbens, deep cerebellar nuclei, and hypothalamus [4]. Brown et al. found similar differences with regard to the atrophy of hypothalamic nuclei, but they also showed that a lower whole hypothalamus volume was significantly associated with higher body mass index in Prader–Willi syndrome [5]. They described that an increased preoccupation with food was associated with lower volumes of the bilateral posterior nuclei and left tubular superior nucleus of the hypothalamus [5].

In addition, there is a large body of case-control studies assessing components of the limbic system in children in the context of different conditions. Several studies have assessed the hippocampus [6,7,8,9,10,11,12,13,14], amygdala [6,14,15], cingulate gyrus [16,17,18], and hypothalamus [4,5]. However, these studies include only a limited number of healthy children, and do not report the sex-specific change of volume across the entire pediatric age span. Volumetric measures of limbic system components across the pediatric age range can be valuable for comparative studies when normal data is scarce. They can also be used for treatment planning and monitoring. In order to provide this much needed reference data, we aimed to assess both age- and sex-related differences of limbic system structures in childhood.

## 2. Materials and Methods

### 2.1. Study Population

This retrospective study was approved by our institutional research ethics board. Informed consent was waived due to the retrospective nature of the study. Patients were identified from the electronic health record data base from January 2010 to December 2019. Patient inclusion criteria were the following: (1) 0–18 years of age, (2) availability of a brain MRI with an isotropic three-dimensional (3D) T1-weighted sequence, (3) normal MR imaging examination of the brain reported by a pediatric neuroradiologist, and (4) absence of neurological disorders. Exclusion criteria were (1) motion artifacts on the 3D T1-weighted sequence and (2) failure of the regions-of-interests (ROIs) on the volumetric analysis to align to the structural MRI on visual inspection.

### 2.2. MRI Acquisition

All included children underwent brain MR imaging at 1.5T or 3T across two scanner vendors (Achieva, Philips Healthcare, Best, The Netherlands; Magnetom Skyra, Siemens Healthineers, Erlangen, Germany) with a dedicated head-coil. High spatial resolution volumetric T1-weighted 3D gradient echo images were acquired either in the sagittal or the axial plane: TR/TE, 6–9/2–5 ms; flip angle: 10–15; resolution isotropic 0.9–1.1 mm; field of view (FOV): 22–24 cm; 150–200 slices.

### 2.3. FreeSurfer Analysis and Visual Inspection

**Figure 1:**
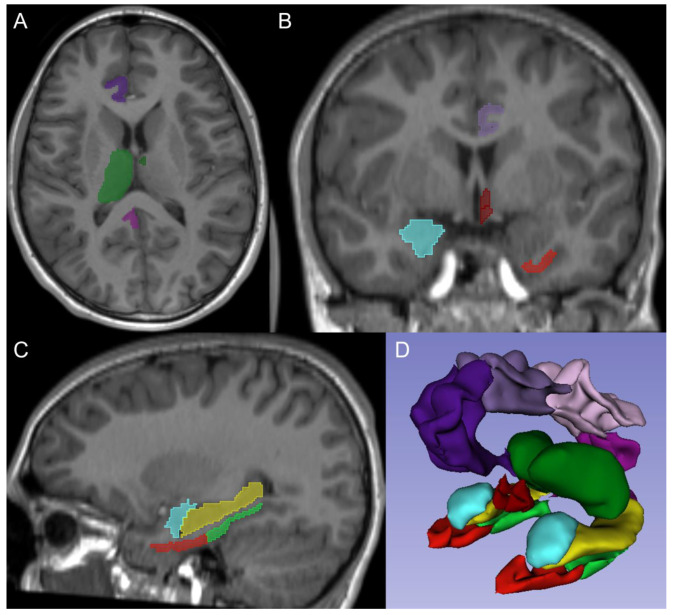
Regions of interest overlay. (**A**–**C**) demonstrate axial, coronal, and sagittal T1-weighted MR images with right cingulate gyrus (pink and purple), whole right thalamus (green), and left antero-ventral nucleus of the thalamus (dark green) in (**A**); right amygdala (light blue), left hypothalamus (dark red), left cingulate gyrus (purple), and left entorhinal cortex (light red) in (**B**); and left amygdala (light blue), left entorhinal cortex (dark red), left parahippocampal cortex (green), and left hippocampus (yellow) in (**C**). (**D**) shows a 3D reformat of the regions of interest using the same color coding as (**A**–**C**).

FreeSurfer is a widely used open-access software package for structural brain imaging analysis [19,20], serving as an automated reconstruction pipeline for the processing of brain MR images including skull stripping, motion artifact correction, B1 bias field correction, gray-white matter segmentation, and region labeling on the cortical surface using different atlases [21,22,23,24,25]. The technical details have been described in previous publications [26,27,28]. De-identified isotropic T1-weighted structural MRI sequences were made available from our local picture archiving and communication system (PACS) and processed using FreeSurfer version 7.3 (Massachusetts General Hospital, Harvard Medical School; http://surfer.nmr.mgh.harvard.edu (accessed on 1 November 2022)). The anatomical ROIs included total brain volume [25], hippocampus [29], parahippocampal gyrus [24], amygdala [30], hypothalamus [21], cingulate gyrus and entorhinal cortex [24], and anteroventral thalamic nucleus and whole thalamus [23]. The parcellated individual neuroanatomical masks corresponding to the ROIs were overlayed to the 3D T1-weighted MRI sequence. All of the structures were visually inspected for accuracy of alignment between the mask and the MRI (Figure 1), which could possibly result in erroneous volume calculation. To ensure the best possible quality of volumetric measurements, patients were excluded from further analysis, if there was a failure of alignment of ROIs to MRI.

### 2.4. Statistical Analysis

Continuous data are provided as mean and standard deviation (SD) and as mean and 95% confidence intervals (Supplemental Table S1: Age- and sex specific confidence intervals of regions of interest). Categorical data are presented as integers and percentages (%). Quadratic trendlines are added to age- and sex-specific data plots (Supplemental Figures S1–S9). Data were plotted using Microsoft Excel ^®^ 2021.

## 3. Results

### 3.1. Study Population and MRI Field Strength

We identified 1100 children fulfilling the inclusion criteria. Of these, 596 (54.2%) were female. Mean age at time of MRI was 10.3 ± 4.5 years (range: 0.67 to 18.2 years). After visual assessment of the segmentations, 376 children (34%) were excluded due motion degradation resulting in suboptimal accuracy of the segmentations. Therefore, the final study cohort consisted of 724 children (401 female, 55.4%), with a mean age at time of MRI of 10.9 ± 4.2 years (range: 1.9 to 18.2 years). The mean age of males was 10 ± 4.1 years (range: 1.9 to 17.9 years). The mean age of females was 11.6 ± 4.1 years (range: 2 to 18.2 years). In total, 493 children were scanned on a 3T MRI scanner (68.1%). Out of 401 female participants, 283 (70.6%) were imaged using a 3T MRI scanner, and out of 323 male participants, 210 (65%) were scanned on a 3T MRI scanner. Demographic information is summarized in Table 1.

### 3.2. Volumetric Results

Total brain volume and left and right hippocampus, parahippocampal gyrus, amygdala, hypothalamus, cingulate gyrus, entorhinal cortex, anteroventral thalamic nucleus, and whole thalamus are plotted as a function of age and sex (Supplemental Figures S1–S9). Age- and sex-specific mean volumes with standard deviations of the ROIs are provided in Table 2. Age- and sex-specific mean volumes and confidence intervals are provided in the Supplemental Table S1. Results are provided in brackets by age in years. Number of female patients in age brackets are: 9 for 2–3 years, 13 for 3–4 years, 16 for 4–5 years, 13 for 5–6 years, 14 for 6–7 years, 20 for 7–8 years, 24 for 8–9 years, 25 for 9–10 years, 24 for 10–11 years, 33 for 11–12 years, 29 for 12–13 years, 32 for 13–14 years, 45 for 14–15 years, 50 for 15–16 years, 26 for 16–17 years, and 28 for 17–18 years. Number of male patients in age brackets are: 8 for 2–3 years, 18 for 3–4 years, 15 for 4–5 years, 22 for 5–6 years, 30 for 6–7 years, 22 for 7–8 years, 27 for 8–9 years, 10 for 9–10 years, 30 for 10–11 years, 25 for 11–12 years, 28 for 12–13 years, 23 for 13–14 years, 20 for 14–15 years, 14 for 15–16 years, 21 for 16–17 years, and 10 for 17–18 years.

*Total brain volume:* For females, the total brain volume increased from 955 ± 70 mL at the age of 2–3 years to 1140 ± 110 mL at the age of 17–18 years. Similarly, the total brain volume increased for males from 1004 ± 83 mL to 1263 ± 96 mL. The maximum volume was noted at 11–12 years for females (1188 ± 90 mL) and at 14–15 years for males (1310 ± 159 mL).

*Hippocampus:* For females, the hippocampus increased from 2.59 ± 0.43 mL on the left and 2.59 ± 0.18 mL on the right at the age of 2–3 years to 3.36 ± 0.39 mL on the left and 3.43 ± 0.37 mL on the right at the age of 17–18 years, which is equivalent to an increase of 22.9% and 24.5%, respectively. For males, the hippocampus increased from 2.67 ± 0.29 mL on the left and 2.81 ± 0.26 mL on the right at the age of 2–3 years to 3.64 ± 0.38 mL on the left and 3.76 ± 0.37 mL on the right at the age of 17–18 years, which is equivalent to an increase of 26.6% and 25.3%, respectively. The maximum volume was noted for females on the left and right at 16–17 years (3.49 ± 0.32 mL and 3.52 ± 0.33 mL, respectively). The maximum volume was noted for males on the left at 14–15 years (3.77 ± 0.47 mL) and on the right at 16–17 years (3.83 ± 0.45 mL).

*Parahippocampal gyrus:* For females, the parahippocampal gyrus increased from 1.93 ± 0.26 mL on the left and 1.79 ± 0.24 mL on the right at the age of 2–3 years to 2.33 ± 0.29 mL on the left and 2.06 ± 0.2 mL on the right at the age of 17–18 years, which is equivalent to an increase of 17.2% and 13.1%, respectively. For males, the parahippocampal gyrus increased from 2.04 ± 0.16 mL on the left and 1.71 ± 0.23 mL on the right at the age of 2–3 years to 2.33 ± 0.27 mL on the left and 2.19 ± 0.32 mL on the right at the age of 17–18 years, which is equivalent to an increase of 12.4% and 22%, respectively. The maximum volume was noted for females on the left at 10–11 years (2.48 ± 0.42 mL) and on the right at 11–12 years (2.23 ± 0.28 mL). The maximum volume was noted for males on the left and on the right at 14–15 years (2.49 ± 0.36 mL and 2.38 ± 0.39 mL, respectively).

*Amygdala:* For females, the amygdala increased from 1.37 ± 0.23 mL on the left and 1.34 ± 0.1 mL on the right at the age of 2–3 years to 1.65 ± 0.17 mL on the left and 1.72 ± 0.18 mL on the right at the age of 17–18 years, which is equivalent to an increase of 17% and 22.1%, respectively. For males, the amygdala increased from 1.4 ± 0.16 mL on the left and 1.43 ± 0.16 mL on the right at the age of 2–3 years to 1.87 ± 0.21 mL on the left and 1.95 ± 0.22 mL on the right at the age of 17–18 years, which is equivalent to an increase of 25.1% and 26.7%, respectively. The maximum volume was noted for females on the left and on the right at 16–17 years (1.75 ± 0.17 mL and 1.84 ± 0.18 mL, respectively). The maximum volume was noted for males on the left and on the right at 15–16 years (1.93 ± 0.25 mL and 1.97 ± 0.22 mL, respectively).

*Hypothalamus:* For females, the hypothalamus increased from 0.3 ± 0.02 mL on the left and 0.3 ± 0.03 mL on the right at the age of 2–3 years to 0.41 ± 0.04 mL on the left and 0.4 ± 0.04 mL on the right at the age of 17–18 years, which is equivalent to an increase of 26.8% and 25%, respectively. For males, the hypothalamus increased from 0.32 ± 0.05 mL on the left and 0.3 ± 0.04 mL on the right at the age of 2–3 years to 0.46 ± 0.04 mL on the left and 0.44 ± 0.03 mL on the right at the age of 17–18 years, which is equivalent to an increase of 30.4% and 31.8%, respectively. The maximum volume was noted for females on the left and on the right at 15–16 years (0.41 ± 0.03 mL and 0.4 ± 0.03 mL, respectively). The maximum volume was noted for males on the left and on the right at 17–18 years (0.46 ± 0.04 mL and 0.44 ± 0.03 mL, respectively).

*Cingulate gyrus:* For females, the cingulate gyrus increased from 11.69 ± 1.32 mL on the left and 10.42 ± 1.03 mL on the right at the age of 2–3 years to 12.96 ± 1.55 mL on the left and 10.71 ± 1.32 mL on the right at the age of 17–18 years, which is equivalent to an increase of 9.8% and 2.7%, respectively. For males, the cingulate gyrus increased from 12.93 ± 1.57 mL on the left and 10.78 ± 1.28 mL on the right at the age of 2–3 years to 14.41 ± 2.19 mL on the left and 12.36 ± 1.45 mL on the right at the age of 17–18 years, which is equivalent to an increase of 10.3% and 12.8%, respectively. The maximum volume was noted for females on the left at 11–12 years (14.44 ± 1.82 mL) and on the right at 10–11 years (12.15 ± 1.17 mL). The maximum volume was noted for males on the left and on the right at 8–9 years (15.75 ± 2.18 mL and 13.24 ± 2.04 mL, respectively).

*Entorhinal cortex:* For females, the entorhinal cortex increased from 1.17 ± 0.25 mL on the left and 1.19 ± 0.14 mL on the right at the age of 2–3 years to 1.75 ± 0.31 mL on the left and 1.61 ± 0.31 mL on the right at the age of 17–18 years, which is equivalent to an increase of 33.1% and 26.1%, respectively. For males, the entorhinal cortex increased from 1.41 ± 0.41 mL on the left and 1.39 ± 0.26 mL on the right at the age of 2–3 years to 1.81 ± 0.32 mL on the left and 1.74 ± 0.59 mL on the right at the age of 17–18 years, which is equivalent to an increase of 22.1% and 20.1%, respectively. The maximum volume was noted for females on the left at 16–17 years (1.78 ± 0.41 mL) and on the right at 13–14 years (1.64 ± 0.46 mL). The maximum volume was noted for males on the left and on the right at 9–10 years (2 ± 0.36 mL and 2 ± 0.58 mL, respectively).

*Anteroventral thalamic nucleus:* For females, the anteroventral thalamic nucleus increased from 0.13 ± 0.01 mL on the left and 0.14 ± 0.02 mL on the right at the age of 2–3 years to 0.14 ± 0.02 mL on the left and 0.15 ± 0.02 mL on the right at the age of 17–18 years, which is equivalent to an increase of 7.1% and 6.7%, respectively. For males, the anteroventral thalamic nucleus increased from 0.12 ± 0.02 mL on the left and 0.14 ± 0.02 mL on the right at the age of 2–3 years to 0.16 ± 0.03 mL on the left and 0.16 ± 0.03 mL on the right at the age of 17–18 years, which is equivalent to an increase of 25% and 12.5%, respectively. The maximum volume was noted for females on the left at 11–12 years (0.15 ± 0.02 mL) and on the right at 14–15 years (0.16 ± 0.03 mL). The maximum volume was noted for males on the left at 9–10 years (0.16 ± 0.02 mL) and on the right at 5–6 years (0.17 ± 0.02 mL).

*Whole thalamus:* For females, the whole thalamus increased from 5.92 ± 0.4 mL on the left and 6.09 ± 0.39 mL on the right at the age of 2–3 years to 7.16 ± 0.71 mL on the left and 6.87 ± 0.73 mL on the right at the age of 17–18 years, which is equivalent to an increase of 17.3% and 11.4%, respectively. For males, the whole thalamus increased from 6.12 ± 0.61 mL on the left and 6.3 ± 0.62 mL on the right at the age of 2–3 years to 7.97 ± 0.83 mL on the left and 7.94 ± 0.84 mL on the right at the age of 17–18 years, which is equivalent to an increase of 23.2% and 20.7%, respectively. The maximum volume was noted for females on the left at 16–17 years (7.38 ± 0.73 mL) and on the right at 11–12 years (7.32 ± 0.68 mL). The maximum volume was noted for males on the left at 15–16 years (8.16 ± 0.96 mL) and on the right at 17–18 years (7.94 ± 0.84 mL).

## 4. Discussion

This study provides an anatomically detailed analysis of the absolute volumes of the limbic system in childhood. We report age-related trajectories and sex-related differences of the total brain volume and limbic system in a cohort of 724 children. The reference volumes are intended to serve as normal values in comparative studies either in the form of absolute values or percentages of total brain volume.

The volumes of components of the limbic system have been studied in numerous case-control studies in the context of different conditions in childhood including Prader–Willi syndrome [4,5], schizophrenia [11,12,16,18], bipolar disorder [13], depression [14], anxiety [31], autism spectrum disorder [32,33], attention deficit hyperactivity disorder [32,33], and children with obsessive-compulsive disorder [32,33]. However, normal values of the structures of the limbic system across the pediatric age range specific to sex and age are scarce. With regard to previously published normal values, our results are in line with available data. Total brain volume peaked for females at age 11–12 years (1188 ± 90 mL) and for males at age 14–15 years (1310 ± 159 mL), which is in line with prior longitudinal studies in healthy children [34,35,36]. Similarly, the hippocampus growth trajectory is consistent with prior studies, which reported a fast volume increase until 8 to 10 years of life followed by a slower volume increase [37]. The hippocampal volume at 5 years of age is consistent with manual segmentations in a cohort from Finland [38] and the amygdala volume is consistent with a prior study by Zhou and colleagues [39]. Østby et al. reported the thalamic volume in normal developing children at ages 8–11 and 12–15. Their results are comparable to ours between the ages of 11 to 18 years [40]. With regard to the volume of the parahippocampal gyrus, hypothalamus, cingulate gyrus, entorhinal gyrus, and AV-nucleus of the thalamus, there are only very limited data available. While Wu et al. reported normal values of the hypothalamus in healthy and obese children, their study included only 18 children each with an average age of 8.3 ± 0.9 and 9 ± 0.9 years of age, respectively [4]. Although their study cohort is limited, reported hypothalamic volumes are in line with ours. This is in contrast to the analysis by Isiklar and colleagues, who reported the hypothalamic volume in the pediatric age range [41]. The hypothalamic volumes reported in their study are larger than in the study by Wu et al. and ours. This discrepancy is possibly related to the different segmentation pipelines used (MRICloud vs. FreeSurfer) [4,41]. Frisosky Abuaf et al. reported hypothalamic volumes of 15 healthy adults as percentages of total intracranial volume. These volumes are difficult to compare with our results due to the lack of presentation of absolute volumes [42]. Brown et al. reported hypothalamic volumes of 40 healthy adults with a mean age of 22.8 years [5]. Reported volumes are comparable to our volumes at the highest age range, which further highlights the importance of the segmentation pipelines when comparing segmented brain volumes of interest [5]. With regard to the parahippocampal gyrus, cingulate gyrus, entorhinal gyrus, and AV-nucleus of the thalamus, we did not find further studies reporting absolute volumetric values across the pediatric age span.

The limbic system plays an integral role in the regulation of the autonomic nervous system, emotion- and goal-related behavior, feeding behavior, learning, and memory [4,13]. Volumes of limbic system components have been assessed in various conditions. In children with Prader–Willi syndrome, studies have shown a lower volume of the thalamus, hippocampus, amygdala, and hypothalamus, among others [4,5]. In childhood onset schizophrenia, the hippocampus develops a volume deficit that is not apparent during initial assessment but worsens over time [8,10]. In pediatric bipolar disorder, children were shown to have smaller hippocampal volumes compared to typically developing children [13]. High childhood anxiety levels were associated with a significantly enlarged left amygdala volume [31]. In studies comparing attention deficit hyperactivity disorder, autism spectrum disorder, and obsessive-compulsive disorder, children with attention deficit hyperactivity disorder showed significantly smaller hippocampal volume compared to children with obsessive compulsive disorder [33]. In adolescents, patients with restrictive-type anorexia nervosa had a significantly decreased gray matter volume in the bilateral middle cingulate cortex [43]. Similar to the literature in children, several studies assessed the volume of the limbic system in adults in the context of different conditions. The volume of the hippocampus was found to be significantly lower in patients with cluster headache compared to healthy controls [44]. In veterans with post-traumatic stress disorder, the bilateral amygdala and hippocampus were shown to be significantly smaller compared to controls [45]. The hypothalamus was noted to be significantly smaller in women using oral contraceptive pills [46]. In patients with multiple sclerosis, the hypothalamus, hippocampus, and anterior thalamus were significantly smaller compared to healthy controls [42]. In patients with substance addiction, volumetric MRI showed decreased volumes of the anterior cingulate and anterior thalamus [47]. Investigations driven by a volumetric approach provide important insights into the neuroanatomic manifestations of various conditions across the human life span. Quantification of structural abnormalities in brain MRIs may help elucidate new pathological phenotypes of diseases, which so far have not been recognized by visual analysis alone. Determining and characterizing neuroanatomic patterns may provide important pathophysiological insights and aid in the development of diagnostic criteria and predictive biomarkers to identify patients at risk, facilitate the implementation of anatomically targeted therapeutic strategies, and monitor treatment-related changes. In addition, age- and sex-specific measurements of the limbic system help to elucidate the development and maturation of this region.

Our study has limitations that need to be taken into account when interpreting the data. Due to the retrospective nature of the study, there was heterogeneity in the MRI sequence acquisition, including the use of different field strengths and imaging parameters. However, since the heterogeneity in image acquisition reflects clinical practice, age- and sex-specific normal volumes of the total brain volume and limbic system need to incorporate these technical variations. The aim of this study was not to draw conclusions on the variability of the volume regarding age, sex, or handedness, but to serve as a reference for volumes in studies on the neuroanatomic prevalence of pathologies affecting the limbic system.

## 5. Conclusions

This study provides an anatomically detailed analysis of the absolute age- and sex-specific volumes of the limbic system and the total brain volume across the pediatric age span. These reference volumes are intended to serve as normal values in comparative studies either in the form of absolute values or percentages of the total brain volumes. Quantification of structural abnormalities of the limbic system on brain MRI may lead to further insights into phenotypical variations of diseases and may help elucidate new pathological phenotypes of diseases, which have not been recognized by visual analysis alone. Further efforts employing our approach of reporting normal reference values could help to improve the understanding of developmental disorders, traumatic brain injury, and other neurological disorders, including epilepsy, cerebral palsy, and multiple sclerosis. The change of volume of brain structures secondary to interventions and treatments is another avenue of research, where reference volumes are needed to evaluate the therapeutic response and side effects. Volume could serve as a biomarker providing objective measures of response to treatment.

## Figures and Tables

**Table 1:** Demographics of the children included in the volumetric data analysis.

		1.5T and 3T MRI (N, %)	3T MRI (N, %)	Mean Age Years (y) ± STD	Age Range Years (y)
**Included**	all	1100, 100%	738, 67.1%	10.3 ± 4.5 y	0.7–18.3 y
	female	596, 54.2%	406, 68.1%	10.9 ± 4.5 y	0.7–18.3 y
	male	504, 45.8%	332, 65.9%	9.69 ± 4.4 y	0.8–17.9 y
**Final cohort**	all	724, 100%	493, 68.1%	10.9 ± 4.2 y	1.9–18.2 y
	female	401, 55.4%	283, 70.6%	11.6 ± 4.1 y	2–18.2 y
	male	323, 44.6%	210, 65%	10 ± 4.1 y	1.9–17.9 y

**Legend:** Number (N), percentage (%), standard deviation (STD).

**Table 2:** Age- and sex-specific mean volumes of regions of interest.

			2–3 y		3–4 y		4–5 y		5–6 y		6–7 y		7–8 y		8–9 y		9–10 y		10–11 y		11–12 y		12–13 y		13–14 y		14–15 y		15–16 y		16–17 y		17–18 y	
			*Mean*	*STD*	*Mean*	*STD*	*Mean*	*STD*	*Mean*	*STD*	*Mean*	*STD*	*Mean*	*STD*	*Mean*	*STD*	*Mean*	*STD*	*Mean*	*STD*	*Mean*	*STD*	*Mean*	*STD*	*Mean*	*STD*	*Mean*	*STD*	*Mean*	*STD*	*Mean*	*STD*	*Mean*	*STD*
**TBV**	*Female*		955	70	1042	84	1080	86	1116	90	1126	88	1115	80	1113	87	1157	159	1182	74	**1188**	**90**	1165	73	1173	79	1163	102	1147	98	1145	118	1140	110
	*Male*		1004	83	1144	118	1172	139	1211	110	1212	106	1235	92	1296	123	1264	104	1271	88	1262	94	1260	104	1271	103	**1310**	**159**	1308	143	1243	81	1263	96
																																		
**HIPPO (×10^−3^)**	*Female*	Left	2.59	0.43	2.82	0.31	2.97	0.24	3.07	0.25	3.06	0.27	3.12	0.22	3.18	0.30	3.31	0.45	3.32	0.29	3.37	0.34	3.34	0.29	3.38	0.27	3.38	0.30	3.35	0.30	**3.49**	**0.32**	3.36	0.39
		Right	2.59	0.18	2.94	0.32	3.03	0.27	3.16	0.29	3.15	0.29	3.21	0.24	3.22	0.29	3.36	0.44	3.36	0.23	3.42	0.38	3.40	0.28	3.42	0.29	3.43	0.32	3.41	0.32	**3.52**	**0.33**	3.43	0.37
	*Male*	Left	2.67	0.29	3.02	0.34	3.14	0.31	3.25	0.27	3.22	0.33	3.46	0.49	3.44	0.47	3.66	0.31	3.57	0.40	3.38	0.33	3.51	0.38	3.58	0.31	**3.77**	**0.47**	3.60	0.40	3.68	0.45	3.64	0.38
		Right	2.81	0.26	3.10	0.38	3.19	0.31	3.37	0.29	3.27	0.31	3.48	0.32	3.54	0.44	3.76	0.34	3.63	0.43	3.45	0.30	3.64	0.46	3.68	0.34	3.82	0.49	3.69	0.36	**3.83**	**0.45**	3.76	0.37
																																		
**P-HIPPO (×10^−3^)**	*Female*	Left	1.93	0.26	2.20	0.29	2.21	0.27	2.22	0.30	2.37	0.32	2.40	0.40	2.35	0.35	2.29	0.40	**2.48**	**0.42**	2.41	0.31	2.31	0.33	2.38	0.31	2.34	0.37	2.34	0.33	2.37	0.30	2.33	0.29
		Right	1.79	0.24	2.02	0.26	2.08	0.20	2.07	0.23	2.12	0.35	2.21	0.23	2.14	0.39	2.06	0.34	2.23	0.36	**2.23**	**0.28**	2.20	0.31	2.22	0.27	2.14	0.24	2.14	0.21	2.19	0.28	2.06	0.20
	*Male*	Left	2.04	0.16	2.16	0.31	2.20	0.25	2.35	0.37	2.31	0.34	2.45	0.27	2.35	0.37	2.44	0.38	2.43	0.42	2.33	0.37	2.41	0.38	2.49	0.27	**2.49**	**0.36**	2.48	0.29	2.46	0.43	2.33	0.27
		Right	1.71	0.23	2.09	0.33	2.01	0.30	2.14	0.30	2.07	0.30	2.16	0.34	2.20	0.28	2.36	0.37	2.19	0.33	2.17	0.23	2.27	0.35	2.27	0.24	**2.38**	**0.39**	2.35	0.29	2.20	0.45	2.19	0.32
																																		
**AMYG (×10^−3^)**	*Female*	Left	1.37	0.23	1.40	0.11	1.50	0.12	1.52	0.13	1.56	0.14	1.60	0.09	1.57	0.13	1.66	0.23	1.65	0.13	1.70	0.17	1.68	0.13	1.69	0.16	1.69	0.12	1.69	0.16	**1.75**	**0.17**	1.65	0.17
		Right	1.34	0.10	1.44	0.11	1.52	0.14	1.57	0.10	1.58	0.12	1.67	0.13	1.62	0.14	1.71	0.24	1.72	0.13	1.77	0.18	1.72	0.14	1.75	0.15	1.75	0.14	1.75	0.17	**1.84**	**0.18**	1.72	0.18
	*Male*	Left	1.40	0.16	1.55	0.17	1.63	0.20	1.65	0.15	1.65	0.14	1.79	0.25	1.80	0.24	1.79	0.13	1.79	0.17	1.78	0.16	1.84	0.18	1.82	0.17	1.90	0.27	**1.93**	**0.25**	1.88	0.21	1.87	0.21
		Right	1.43	0.16	1.62	0.19	1.65	0.22	1.73	0.16	1.69	0.15	1.78	0.18	1.86	0.28	1.87	0.16	1.85	0.20	1.83	0.18	1.90	0.21	1.90	0.18	1.95	0.24	**1.97**	**0.22**	1.97	0.20	1.95	0.22
																																		
**HYPOTH (×10^−3^)**	*Female*	Left	0.30	0.02	0.34	0.04	0.34	0.04	0.33	0.06	0.32	0.08	0.35	0.04	0.32	0.07	0.37	0.03	0.37	0.05	0.39	0.04	0.39	0.04	0.40	0.03	0.41	0.04	**0.41**	**0.03**	0.41	0.03	0.41	0.04
		Right	0.30	0.02	0.33	0.04	0.33	0.04	0.32	0.07	0.32	0.07	0.36	0.04	0.33	0.06	0.37	0.04	0.37	0.04	0.36	0.05	0.39	0.04	0.39	0.05	0.40	0.05	**0.40**	**0.03**	0.40	0.03	0.40	0.04
	*Male*	Left	0.32	0.05	0.37	0.04	0.35	0.05	0.37	0.04	0.36	0.07	0.35	0.08	0.38	0.07	0.35	0.05	0.40	0.04	0.41	0.04	0.43	0.03	0.44	0.04	0.44	0.05	0.44	0.03	0.42	0.04	**0.46**	**0.04**
		Right	0.30	0.04	0.35	0.04	0.33	0.05	0.36	0.04	0.34	0.08	0.36	0.07	0.36	0.09	0.35	0.07	0.39	0.05	0.40	0.05	0.41	0.03	0.42	0.06	0.43	0.05	0.42	0.04	0.40	0.06	**0.44**	**0.03**
																																		
**CING (×10^−3^)**	*Female*	Left	11.69	1.32	13.12	1.31	13.73	1.16	14.08	1.23	14.16	1.33	13.89	1.52	14.07	1.89	14.12	2.95	14.20	1.65	**14.44**	**1.82**	14.02	1.42	14.21	1.48	13.52	1.57	13.25	1.81	13.83	1.65	12.96	1.55
		Right	10.42	1.03	10.97	1.80	12.33	1.52	12.33	1.51	11.83	1.33	12.14	1.31	11.64	1.72	11.86	2.38	**12.15**	**1.17**	12.05	1.62	12.05	1.43	11.86	1.33	11.34	1.19	11.21	1.51	11.58	1.38	10.71	1.32
	*Male*	Left	12.93	1.57	14.74	1.38	14.48	1.98	15.40	1.79	15.23	1.72	15.33	1.90	**15.75**	**2.18**	15.02	1.28	15.58	1.85	14.88	1.46	14.95	2.24	14.77	1.51	15.95	2.48	15.67	1.88	14.16	1.42	14.41	2.19
		Right	10.78	1.28	12.42	1.38	12.15	1.90	12.81	1.83	12.70	1.70	12.78	1.28	**13.24**	**2.04**	12.87	1.50	12.78	1.42	12.86	1.24	12.44	1.84	12.58	1.80	13.12	1.78	12.85	1.95	11.32	1.24	12.36	1.45
																																		
**ENTO (×10^−3^)**	*Female*	Left	1.17	0.25	1.53	0.47	1.56	0.38	1.56	0.45	1.55	0.22	1.57	0.33	1.49	0.29	1.58	0.34	1.77	0.40	1.63	0.23	1.65	0.35	1.69	0.49	1.67	0.48	1.74	0.48	**1.78**	**0.41**	1.75	0.31
		Right	1.19	0.14	1.47	0.36	1.56	0.39	1.46	0.35	1.46	0.24	1.54	0.37	1.46	0.26	1.46	0.24	1.62	0.39	1.56	0.45	1.52	0.23	**1.64**	**0.46**	1.59	0.33	1.54	0.30	1.55	0.24	1.61	0.31
	*Male*	Left	1.41	0.41	1.65	0.47	1.51	0.31	1.71	0.41	1.71	0.31	1.85	0.44	1.78	0.34	**2.00**	**0.36**	1.93	0.60	1.84	0.43	1.79	0.48	1.95	0.68	1.92	0.31	1.82	0.27	1.78	0.37	1.81	0.32
		Right	1.39	0.26	1.44	0.33	1.53	0.37	1.69	0.46	1.58	0.33	1.59	0.40	1.67	0.37	**2.00**	**0.58**	1.72	0.46	1.67	0.36	1.66	0.45	1.64	0.37	1.79	0.26	1.69	0.31	1.70	0.32	1.74	0.59
																																		
**AV-THAL (×10^−3^)**	*Female*	Left	0.13	0.01	0.13	0.02	0.15	0.02	0.14	0.02	0.13	0.01	0.15	0.02	0.14	0.02	0.14	0.04	0.14	0.02	**0.15**	**0.02**	0.15	0.02	0.14	0.02	0.15	0.02	0.14	0.02	0.14	0.02	0.14	0.02
		Right	0.14	0.02	0.14	0.02	0.15	0.02	0.15	0.02	0.15	0.02	0.16	0.02	0.15	0.02	0.15	0.04	0.15	0.02	0.16	0.02	0.16	0.02	0.15	0.02	**0.16**	**0.03**	0.15	0.02	0.15	0.02	0.15	0.02
	*Male*	Left	0.12	0.02	0.15	0.02	0.15	0.03	0.16	0.02	0.16	0.02	0.16	0.02	0.16	0.02	**0.16**	**0.02**	0.16	0.02	0.16	0.02	0.15	0.02	0.16	0.02	0.16	0.02	0.16	0.03	0.15	0.02	0.16	0.03
		Right	0.14	0.02	0.16	0.02	0.16	0.03	**0.17**	**0.02**	0.17	0.03	0.16	0.02	0.17	0.02	0.17	0.01	0.17	0.02	0.17	0.02	0.16	0.02	0.17	0.02	0.17	0.03	0.17	0.02	0.16	0.02	0.16	0.03
																																		
**THAL (×10^−3^)**	*Female*	Left	5.92	0.40	6.32	0.44	6.67	0.43	6.82	0.48	6.79	0.50	7.00	0.45	6.79	0.67	7.20	1.26	7.16	0.52	7.36	0.63	7.19	0.61	7.36	0.66	7.25	0.75	7.14	0.71	**7.38**	**0.73**	7.16	0.71
		Right	6.09	0.39	6.38	0.43	6.70	0.38	6.80	0.57	6.85	0.54	6.93	0.52	6.75	0.69	7.17	1.18	7.04	0.42	**7.32**	**0.68**	7.10	0.60	7.24	0.53	7.16	0.68	7.13	0.62	7.27	0.72	6.87	0.73
	*Male*	Left	6.12	0.61	7.10	0.72	7.03	0.79	7.43	0.65	7.41	0.67	7.36	0.47	7.70	0.75	7.73	0.62	7.82	0.62	7.62	0.69	7.78	0.58	7.91	0.69	8.13	0.90	**8.16**	**0.96**	7.71	0.67	7.97	0.83
		Right	6.30	0.62	6.99	0.56	6.99	0.73	7.37	0.70	7.29	0.70	7.25	0.63	7.60	0.75	7.62	0.41	7.70	0.62	7.48	0.58	7.64	0.59	7.78	0.63	7.77	0.80	7.86	0.88	7.57	0.53	**7.94**	**0.84**

**Legend:** Results are given in mean volume in milliliters and standard deviation (STD). Years (y), total brain volume (TBV), hippocampus (HIPPO), parahippocampal gyrus (P-HIPPO), amgydala (AMYG), hypothalamus (HYPOTH), parahippocampal gyrus (P-HIPPO), amygdala (AMYG), hypothalamus (HYPOTH), cingulate gyrus (CING), antero-ventral nucleus of thalamus (AV-THAL), whole thalamus (THAL). The maximum volumes are in bold and underlined.

## Data Availability

The datasets generated during and/or analyzed during the current study are available from the corresponding author on reasonable request.

## Supplementary Materials

The following supporting information can be downloaded at: https://www.mdpi.com/article/10.3390/children10030477/s1, Table S1: Age- and sex specific confidence intervals of regions of interest. Figures S1–S9: Quadratic trendlines are added to age- and sex-specific data plots.
