# Supplementary material for: MRI Volumetric Analysis of the Hypothalamus and Limbic System across the Pediatric Age Span
Source: Children (Basel). 2023 Feb 27;10(3):477. doi: 10.3390/children10030477 (PMC10047273; doi:10.3390/children10030477)
Supplement: Supplementary file 1 [file children-10-00477-s001.zip › children-2244551-supplementary/Table S1.pdf]

Supplemental Table S1. Age- and sex specific confidence intervals of regions of interest

			2-3y			3-4y			4-5y			5-6y			6-7y			7-8y			8-9y			9-10y		
			Lower CI	*Mean*	*Upper CI*	*Lower CI*	*Mean*	*Upper CI*	*Lower CI*	*Mean*	*Upper CI*	*Lower CI*	*Mean*	*Upper CI*	*Lower CI*	*Mean*	*Upper CI*	*Lower CI*	*Mean*	*Upper CI*	*Lower CI*	*Mean*	*Upper CI*	*Lower CI*	*Mean*	*Upper CI*
**TBV**	*F*		908.84	954.76	1000.67	996.60	1042.49	1088.39	1037.73	1079.93	1122.13	1066.93	1115.87	1164.81	1080.21	1126.16	1172.11	1080.16	1115.12	1150.09	1078.29	1113.06	1147.83	1094.51	1156.80	1219.09
	M		946.78	1004.01	1061.25	1089.53	1144.18	1198.84	1101.65	1172.02	1242.39	1165.05	1211.00	1256.95	1174.44	1212.43	1250.42	1196.23	1234.72	1273.20	1249.59	1296.16	1342.74	1199.65	1264.12	1328.60
**HIPPO ($x10^{-3}$)**	*F*	L	2.31	2.59	2.87	2.65	2.82	2.98	2.85	2.97	3.09	2.93	3.07	3.20	2.92	3.06	3.21	3.02	3.12	3.22	3.06	3.18	3.29	3.13	3.31	3.48
		R	2.47	2.59	2.71	2.77	2.94	3.12	2.90	3.03	3.17	3.00	3.16	3.32	3.00	3.15	3.30	3.11	3.21	3.32	3.10	3.22	3.33	3.19	3.36	3.54
	M	L	2.47	2.67	2.87	2.86	3.02	3.17	2.98	3.14	3.30	3.13	3.25	3.36	3.10	3.22	3.34	3.26	3.46	3.66	3.27	3.44	3.62	3.47	3.66	3.85
		R	2.63	2.81	2.99	2.92	3.10	3.27	3.03	3.19	3.34	3.25	3.37	3.49	3.16	3.27	3.37	3.35	3.48	3.61	3.38	3.54	3.71	3.55	3.76	3.97
**P-HIPPO ($x10^{-3}$)**	*F*	L	1.76	1.93	2.10	2.04	2.20	2.35	2.07	2.21	2.34	2.06	2.22	2.39	2.20	2.37	2.54	2.22	2.40	2.57	2.20	2.35	2.49	2.13	2.29	2.45
		R	1.63	1.79	1.95	1.88	2.02	2.16	1.98	2.08	2.18	1.95	2.07	2.19	1.93	2.12	2.30	2.11	2.21	2.31	1.98	2.14	2.29	1.93	2.06	2.20
	M	L	1.93	2.04	2.15	2.02	2.16	2.30	2.07	2.20	2.32	2.20	2.35	2.51	2.19	2.31	2.43	2.33	2.45	2.56	2.20	2.35	2.49	2.21	2.44	2.68
		R	1.55	1.71	1.87	1.94	2.09	2.24	1.85	2.01	2.16	2.01	2.14	2.26	1.96	2.07	2.18	2.02	2.16	2.30	2.09	2.20	2.31	2.12	2.36	2.59
**AMYG ($x10^{-3}$)**	*F*	L	1.22	1.37	1.52	1.34	1.40	1.46	1.44	1.50	1.56	1.45	1.52	1.59	1.48	1.56	1.63	1.56	1.60	1.64	1.52	1.57	1.62	1.57	1.66	1.75
		R	1.27	1.34	1.40	1.38	1.44	1.50	1.45	1.52	1.59	1.52	1.57	1.62	1.52	1.58	1.64	1.62	1.67	1.73	1.56	1.62	1.67	1.61	1.71	1.80
	M	L	1.29	1.40	1.51	1.48	1.55	1.63	1.52	1.63	1.73	1.58	1.65	1.71	1.60	1.65	1.70	1.69	1.79	1.90	1.71	1.80	1.90	1.70	1.79	1.87
		R	1.32	1.43	1.54	1.53	1.62	1.71	1.54	1.65	1.76	1.66	1.73	1.80	1.64	1.69	1.75	1.71	1.78	1.85	1.76	1.86	1.97	1.77	1.87	1.97
**HYPOTH ($x10^{-3}$)**	*F*	L	0.29	0.30	0.32	0.31	0.34	0.36	0.32	0.34	0.36	0.30	0.33	0.36	0.28	0.32	0.36	0.33	0.35	0.37	0.29	0.32	0.35	0.36	0.37	0.38
		R	0.29	0.30	0.32	0.31	0.33	0.35	0.31	0.33	0.35	0.29	0.32	0.36	0.28	0.32	0.35	0.34	0.36	0.37	0.31	0.33	0.36	0.36	0.37	0.39
	M	L	0.28	0.32	0.35	0.35	0.37	0.39	0.33	0.35	0.38	0.35	0.37	0.38	0.33	0.36	0.38	0.32	0.35	0.39	0.35	0.38	0.40	0.31	0.35	0.38
		R	0.28	0.30	0.33	0.33	0.35	0.37	0.30	0.33	0.36	0.34	0.36	0.37	0.31	0.34	0.37	0.33	0.36	0.39	0.33	0.36	0.39	0.31	0.35	0.39
**CING ($x10^{-3}$)**	*F*	L	10.83	11.69	12.55	12.41	13.12	13.83	13.16	13.73	14.30	13.41	14.08	14.75	13.46	14.16	14.85	13.22	13.89	14.55	13.31	14.07	14.82	12.96	14.12	15.27
		R	9.75	10.42	11.09	10.00	10.97	11.95	11.59	12.33	13.07	11.51	12.33	13.15	11.13	11.83	12.52	11.56	12.14	12.71	10.95	11.64	12.32	10.93	11.86	12.80
	M	L	11.84	12.93	14.01	14.10	14.74	15.38	13.48	14.48	15.48	14.66	15.40	16.15	14.61	15.23	15.84	14.53	15.33	16.12	14.93	15.75	16.57	14.23	15.02	15.82
		R	9.89	10.78	11.67	11.78	12.42	13.06	11.19	12.15	13.12	12.05	12.81	13.57	12.09	12.70	13.31	12.25	12.78	13.31	12.47	13.24	14.00	11.94	12.87	13.80
**ENTO ($x10^{-3}$)**	*F*	L	1.01	1.17	1.33	1.28	1.53	1.79	1.38	1.56	1.75	1.31	1.56	1.80	1.44	1.55	1.67	1.42	1.57	1.71	1.37	1.49	1.60	1.45	1.58	1.71
		R	1.10	1.19	1.28	1.28	1.47	1.67	1.37	1.56	1.76	1.27	1.46	1.65	1.33	1.46	1.58	1.38	1.54	1.71	1.36	1.46	1.57	1.36	1.46	1.55
	M	L	1.12	1.41	1.70	1.44	1.65	1.87	1.36	1.51	1.67	1.54	1.71	1.88	1.60	1.71	1.82	1.66	1.85	2.03	1.66	1.78	1.91	1.78	2.00	2.23
		R	1.21	1.39	1.57	1.29	1.44	1.60	1.35	1.53	1.72	1.49	1.69	1.88	1.47	1.58	1.70	1.43	1.59	1.76	1.53	1.67	1.81	1.64	2.00	2.36
**AV-THAL ($x10^{-3}$)**	*F*	L	0.12	0.13	0.13	0.12	0.13	0.14	0.14	0.15	0.16	0.13	0.14	0.15	0.13	0.13	0.14	0.14	0.15	0.16	0.13	0.14	0.14	0.13	0.14	0.16
		R	0.12	0.14	0.15	0.13	0.14	0.15	0.14	0.15	0.16	0.14	0.15	0.16	0.14	0.15	0.16	0.15	0.16	0.16	0.14	0.15	0.16	0.14	0.15	0.17
	M	L	0.11	0.12	0.13	0.14	0.15	0.16	0.14	0.15	0.17	0.15	0.16	0.17	0.15	0.16	0.16	0.15	0.16	0.16	0.15	0.16	0.17	0.15	0.16	0.17
		R	0.13	0.14	0.15	0.15	0.16	0.17	0.14	0.16	0.17	0.16	0.17	0.18	0.16	0.17	0.18	0.15	0.16	0.16	0.16	0.17	0.17	0.16	0.17	0.18
**THAL ($x10^{-3}$)**	*F*	L	5.66	5.92	6.18	6.09	6.32	6.56	6.46	6.67	6.88	6.56	6.82	7.08	6.53	6.79	7.05	6.80	7.00	7.20	6.52	6.79	7.06	6.70	7.20	7.69
		R	5.84	6.09	6.35	6.14	6.38	6.61	6.51	6.70	6.88	6.49	6.80	7.11	6.57	6.85	7.13	6.71	6.93	7.16	6.48	6.75	7.03	6.70	7.17	7.63
	M	L	5.70	6.12	6.54	6.77	7.10	7.43	6.62	7.03	7.43	7.16	7.43	7.70	7.17	7.41	7.65	7.16	7.36	7.56	7.41	7.70	7.98	7.34	7.73	8.11
		R	5.87	6.30	6.73	6.73	6.99	7.25	6.62	6.99	7.36	7.07	7.37	7.66	7.04	7.29	7.54	6.99	7.25	7.51	7.32	7.60	7.88	7.37	7.62	7.88

			10-11y			11-12y			12-13y			13-14y			14-15y			15-16y			16-17y			17-18y		
			Lower CI	*Mean*	*Upper CI*	*Lower CI*	*Mean*	*Upper CI*	*Lower CI*	*Mean*	*Upper CI*	*Lower CI*	*Mean*	*Upper CI*	*Lower CI*	*Mean*	*Upper CI*	*Lower CI*	*Mean*	*Upper CI*	*Lower CI*	*Mean*	*Upper CI*	*Lower CI*	*Mean*	*Upper CI*
**TBV**	*F*		1152.81	1182.30	1211.78	1157.44	1188.16	1218.87	1138.35	1164.94	1191.53	1145.00	1172.52	1200.04	1133.16	1162.94	1192.72	1119.77	1146.87	1173.97	1099.67	1145.14	1190.62	1099.59	1140.20	1180.80
	M		1240.02	1271.50	1302.97	1225.48	1262.29	1299.10	1221.06	1259.73	1298.40	1228.79	1270.83	1312.86	1240.43	1309.93	1379.43	1233.36	1308.18	1383.00	1208.57	1243.15	1277.73	1203.17	1262.69	1322.20
**HIPPO ($x10^{-3}$)**	*F*	L	3.21	3.32	3.44	3.25	3.37	3.48	3.23	3.34	3.45	3.29	3.38	3.47	3.29	3.38	3.47	3.27	3.35	3.43	3.36	3.49	3.61	3.22	3.36	3.51
		R	3.27	3.36	3.45	3.29	3.42	3.55	3.30	3.40	3.50	3.32	3.42	3.52	3.34	3.43	3.53	3.32	3.41	3.50	3.40	3.52	3.65	3.29	3.43	3.57
	M	L	3.43	3.57	3.71	3.25	3.38	3.51	3.37	3.51	3.65	3.45	3.58	3.71	3.56	3.77	3.97	3.39	3.60	3.81	3.49	3.68	3.87	3.40	3.64	3.87
		R	3.48	3.63	3.79	3.33	3.45	3.57	3.47	3.64	3.82	3.54	3.68	3.82	3.61	3.82	4.04	3.50	3.69	3.88	3.64	3.83	4.03	3.53	3.76	3.99
**P-HIPPO ($x10^{-3}$)**	*F*	L	2.31	2.48	2.65	2.31	2.41	2.52	2.20	2.31	2.43	2.27	2.38	2.49	2.23	2.34	2.45	2.24	2.34	2.43	2.25	2.37	2.48	2.22	2.33	2.44
		R	2.08	2.23	2.37	2.14	2.23	2.33	2.09	2.20	2.31	2.12	2.22	2.31	2.07	2.14	2.21	2.08	2.14	2.20	2.08	2.19	2.29	1.98	2.06	2.14
	M	L	2.28	2.43	2.58	2.18	2.33	2.47	2.27	2.41	2.55	2.38	2.49	2.60	2.33	2.49	2.65	2.33	2.48	2.63	2.28	2.46	2.64	2.17	2.33	2.50
		R	2.07	2.19	2.31	2.08	2.17	2.26	2.14	2.27	2.40	2.17	2.27	2.37	2.21	2.38	2.55	2.20	2.35	2.50	2.01	2.20	2.39	1.99	2.19	2.39
**AMYG ($x10^{-3}$)**	*F*	L	1.60	1.65	1.70	1.64	1.70	1.76	1.63	1.68	1.73	1.64	1.69	1.75	1.65	1.69	1.72	1.64	1.69	1.73	1.69	1.75	1.82	1.58	1.65	1.71
		R	1.67	1.72	1.78	1.71	1.77	1.83	1.67	1.72	1.77	1.70	1.75	1.81	1.71	1.75	1.79	1.71	1.75	1.80	1.78	1.84	1.91	1.65	1.72	1.79
	M	L	1.73	1.79	1.86	1.72	1.78	1.84	1.77	1.84	1.90	1.75	1.82	1.89	1.78	1.90	2.02	1.79	1.93	2.06	1.79	1.88	1.96	1.74	1.87	2.00
		R	1.78	1.85	1.92	1.76	1.83	1.90	1.82	1.90	1.98	1.82	1.90	1.97	1.85	1.95	2.06	1.85	1.97	2.08	1.89	1.97	2.05	1.81	1.95	2.09
**HYPOTH ($x10^{-3}$)**	*F*	L	0.35	0.37	0.39	0.37	0.39	0.40	0.37	0.39	0.40	0.39	0.40	0.41	0.40	0.41	0.42	0.40	0.41	0.42	0.40	0.41	0.42	0.39	0.41	0.42
		R	0.35	0.37	0.39	0.35	0.36	0.38	0.37	0.39	0.40	0.37	0.39	0.40	0.39	0.40	0.41	0.39	0.40	0.41	0.39	0.40	0.41	0.39	0.40	0.41
	M	L	0.38	0.40	0.41	0.39	0.41	0.43	0.42	0.43	0.44	0.43	0.44	0.46	0.42	0.44	0.46	0.42	0.44	0.45	0.40	0.42	0.44	0.44	0.46	0.49
		R	0.38	0.39	0.41	0.38	0.40	0.42	0.40	0.41	0.42	0.40	0.42	0.44	0.41	0.43	0.45	0.40	0.42	0.45	0.38	0.40	0.43	0.43	0.44	0.46
**CING ($x10^{-3}$)**	*F*	L	13.54	14.20	14.86	13.82	14.44	15.07	13.50	14.02	14.54	13.69	14.21	14.72	13.06	13.52	13.98	12.75	13.25	13.75	13.19	13.83	14.46	12.38	12.96	13.53
		R	11.68	12.15	12.62	11.50	12.05	12.60	11.53	12.05	12.57	11.40	11.86	12.32	11.00	11.34	11.69	10.79	11.21	11.63	11.05	11.58	12.11	10.22	10.71	11.20
	M	L	14.92	15.58	16.25	14.31	14.88	15.46	14.12	14.95	15.78	14.15	14.77	15.38	14.86	15.95	17.03	14.69	15.67	16.65	13.56	14.16	14.77	13.05	14.41	15.77
		R	12.27	12.78	13.29	12.37	12.86	13.35	11.76	12.44	13.12	11.85	12.58	13.32	12.33	13.12	13.90	11.83	12.85	13.87	10.79	11.32	11.85	11.46	12.36	13.25
**ENTO ($x10^{-3}$)**	*F*	L	1.61	1.77	1.93	1.56	1.63	1.71	1.52	1.65	1.78	1.52	1.69	1.86	1.53	1.67	1.81	1.61	1.74	1.87	1.62	1.78	1.94	1.63	1.75	1.86
		R	1.47	1.62	1.78	1.41	1.56	1.71	1.44	1.52	1.61	1.48	1.64	1.80	1.49	1.59	1.69	1.46	1.54	1.62	1.46	1.55	1.65	1.49	1.61	1.72
	M	L	1.71	1.93	2.15	1.67	1.84	2.01	1.61	1.79	1.97	1.67	1.95	2.23	1.79	1.92	2.06	1.68	1.82	1.96	1.62	1.78	1.93	1.61	1.81	2.01
		R	1.56	1.72	1.89	1.53	1.67	1.81	1.50	1.66	1.83	1.49	1.64	1.79	1.67	1.79	1.90	1.52	1.69	1.85	1.56	1.70	1.84	1.37	1.74	2.11
**AV-THAL ($x10^{-3}$)**	*F*	L	0.14	0.14	0.15	0.15	0.15	0.16	0.14	0.15	0.16	0.14	0.14	0.15	0.15	0.15	0.16	0.14	0.14	0.15	0.14	0.14	0.15	0.14	0.14	0.15
		R	0.15	0.15	0.16	0.15	0.16	0.16	0.15	0.16	0.16	0.14	0.15	0.16	0.15	0.16	0.17	0.15	0.15	0.16	0.15	0.15	0.16	0.14	0.15	0.16
	M	L	0.15	0.16	0.17	0.15	0.16	0.17	0.15	0.15	0.16	0.15	0.16	0.17	0.15	0.16	0.17	0.15	0.16	0.17	0.14	0.15	0.17	0.14	0.16	0.17
		R	0.16	0.17	0.18	0.16	0.17	0.17	0.15	0.16	0.17	0.16	0.17	0.18	0.15	0.17	0.18	0.16	0.17	0.18	0.15	0.16	0.17	0.14	0.16	0.18
**THAL ($x10^{-3}$)**	*F*	L	6.96	7.16	7.37	7.15	7.36	7.58	6.97	7.19	7.41	7.14	7.36	7.59	7.04	7.25	7.47	6.94	7.14	7.34	7.10	7.38	7.66	6.90	7.16	7.43
		R	6.87	7.04	7.21	7.09	7.32	7.55	6.88	7.10	7.32	7.06	7.24	7.42	6.96	7.16	7.36	6.95	7.13	7.30	6.99	7.27	7.54	6.60	6.87	7.14
	M	L	7.60	7.82	8.05	7.35	7.62	7.89	7.57	7.78	8.00	7.62	7.91	8.19	7.74	8.13	8.52	7.66	8.16	8.67	7.42	7.71	7.99	7.45	7.97	8.48
		R	7.48	7.70	7.92	7.25	7.48	7.70	7.42	7.64	7.86	7.52	7.78	8.03	7.41	7.77	8.12	7.40	7.86	8.31	7.35	7.57	7.80	7.42	7.94	8.46

Legend: Results are given in mean volume in milliliters and 95% confidence intervals (CI). Years (y), total brain volume (TBV), hippocampus (HIPPO), parahippocampal gyrus (P-HIPPO), amygdala (AMYG), hypothalamus (HYPOTH), cingulate gyrus (CING), antero-ventral nucleus of thalamus (AV-THAL), whole thalamus (THAL).
