# Supplementary figures and images for: MRI Volumetric Analysis of the Hypothalamus and Limbic System across the Pediatric Age Span
Source: Children (Basel). 2023 Feb 27;10(3):477. doi: 10.3390/children10030477 (PMC10047273; doi:10.3390/children10030477)

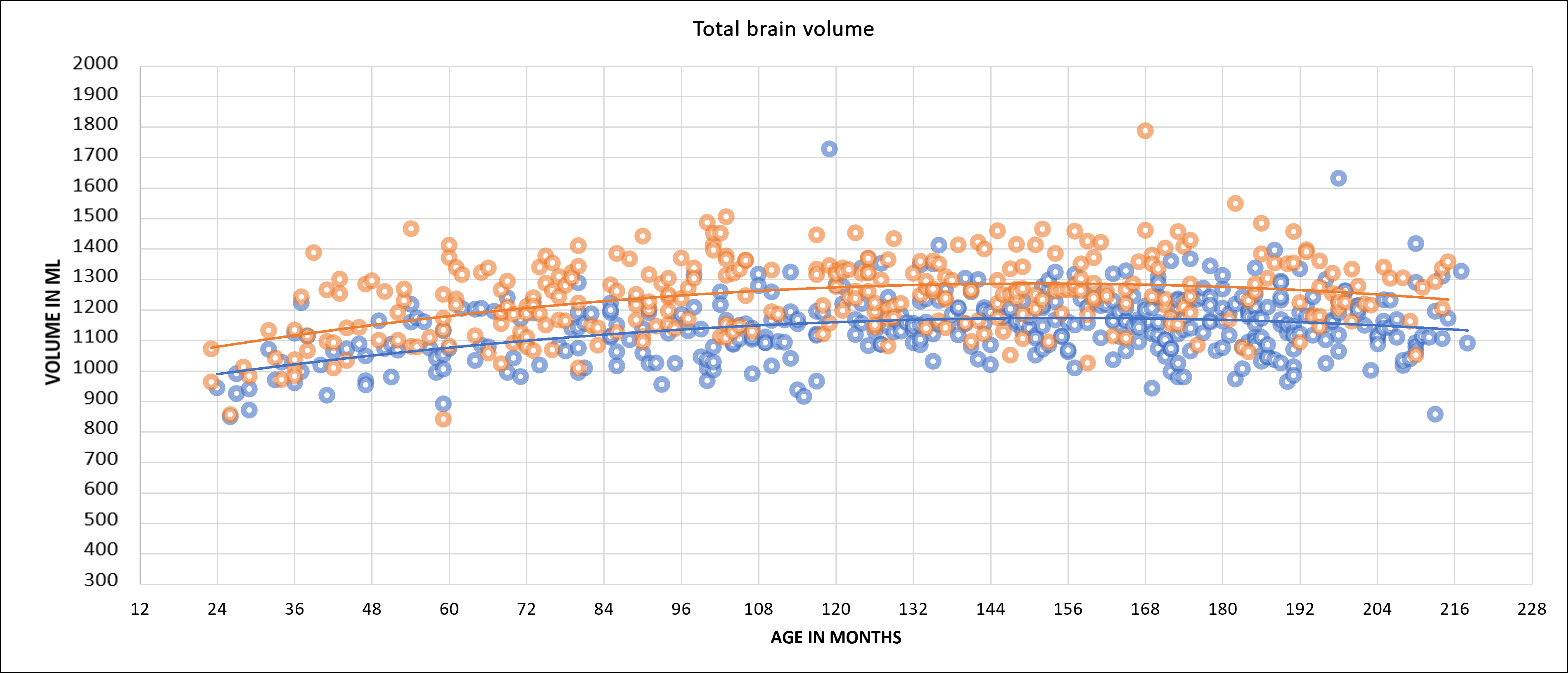

Supplement: Supplementary file 1 [file children-10-00477-s001.zip › children-2244551-supplementary/Figure S1.tif]

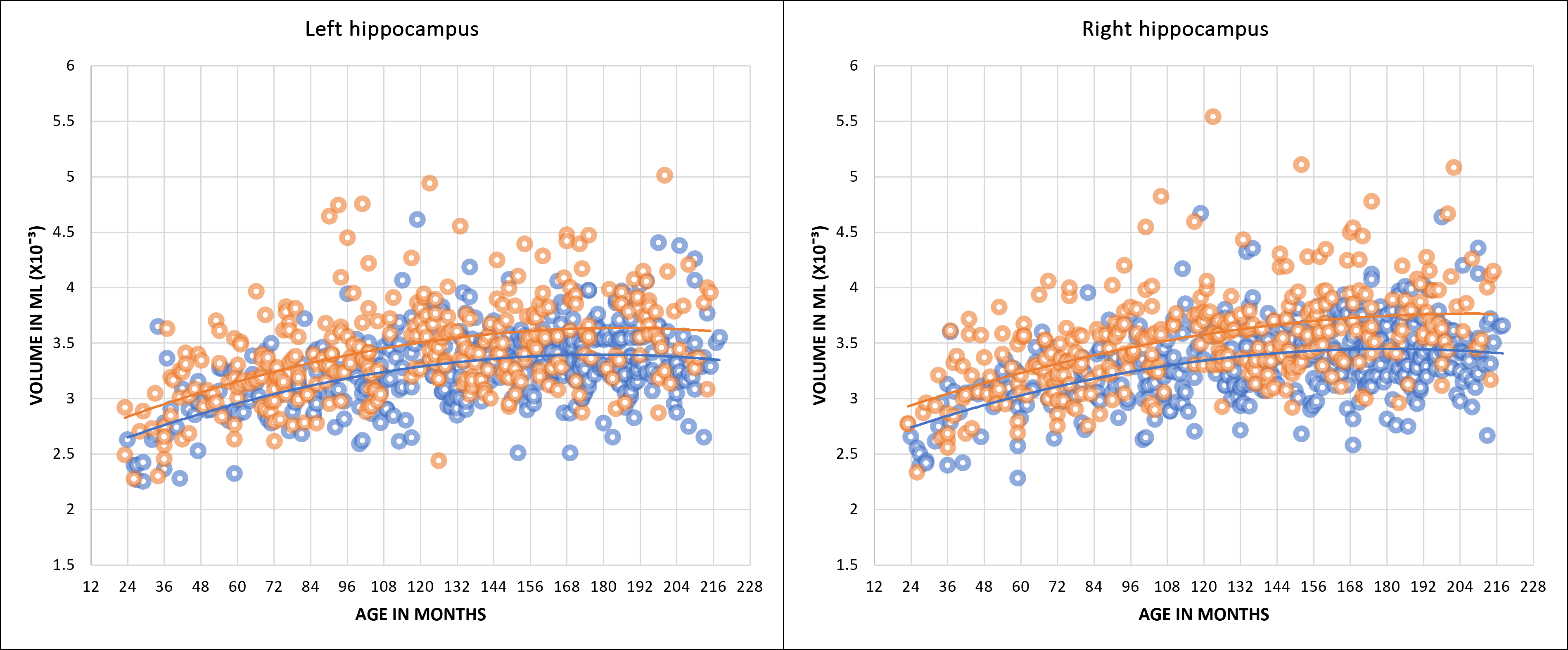

Supplement: Supplementary file 1 [file children-10-00477-s001.zip › children-2244551-supplementary/Figure S2.tif]

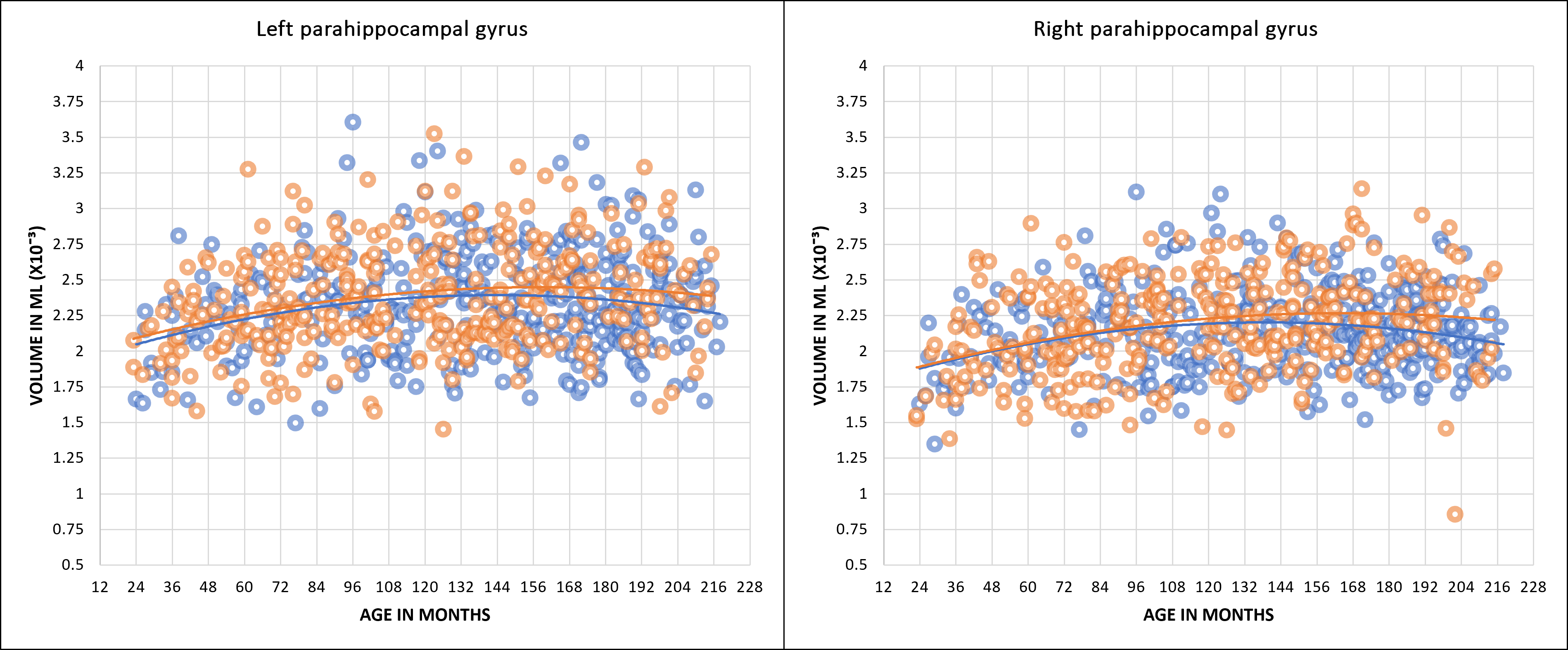

Supplement: Supplementary file 1 [file children-10-00477-s001.zip › children-2244551-supplementary/Figure S3.tif]

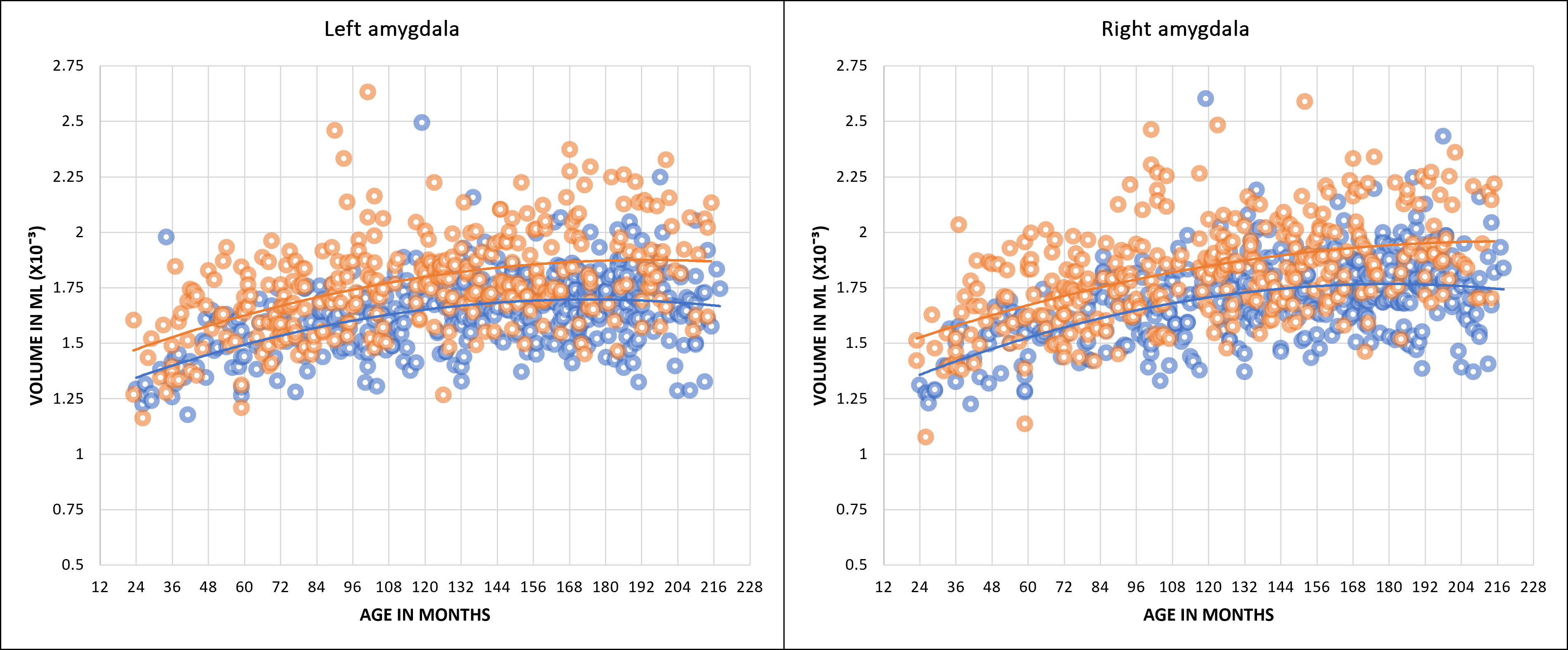

Supplement: Supplementary file 1 [file children-10-00477-s001.zip › children-2244551-supplementary/Figure S4.tif]

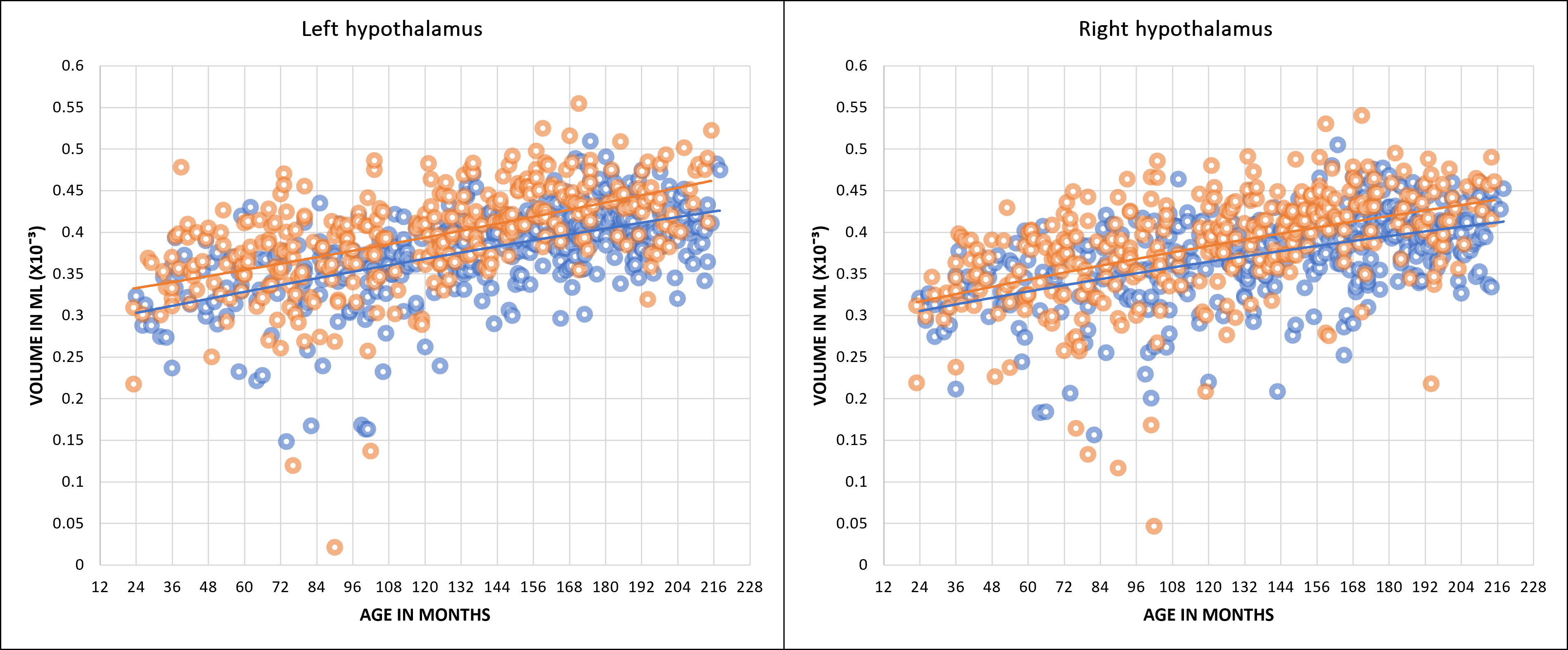

Supplement: Supplementary file 1 [file children-10-00477-s001.zip › children-2244551-supplementary/Figure S5.tif]

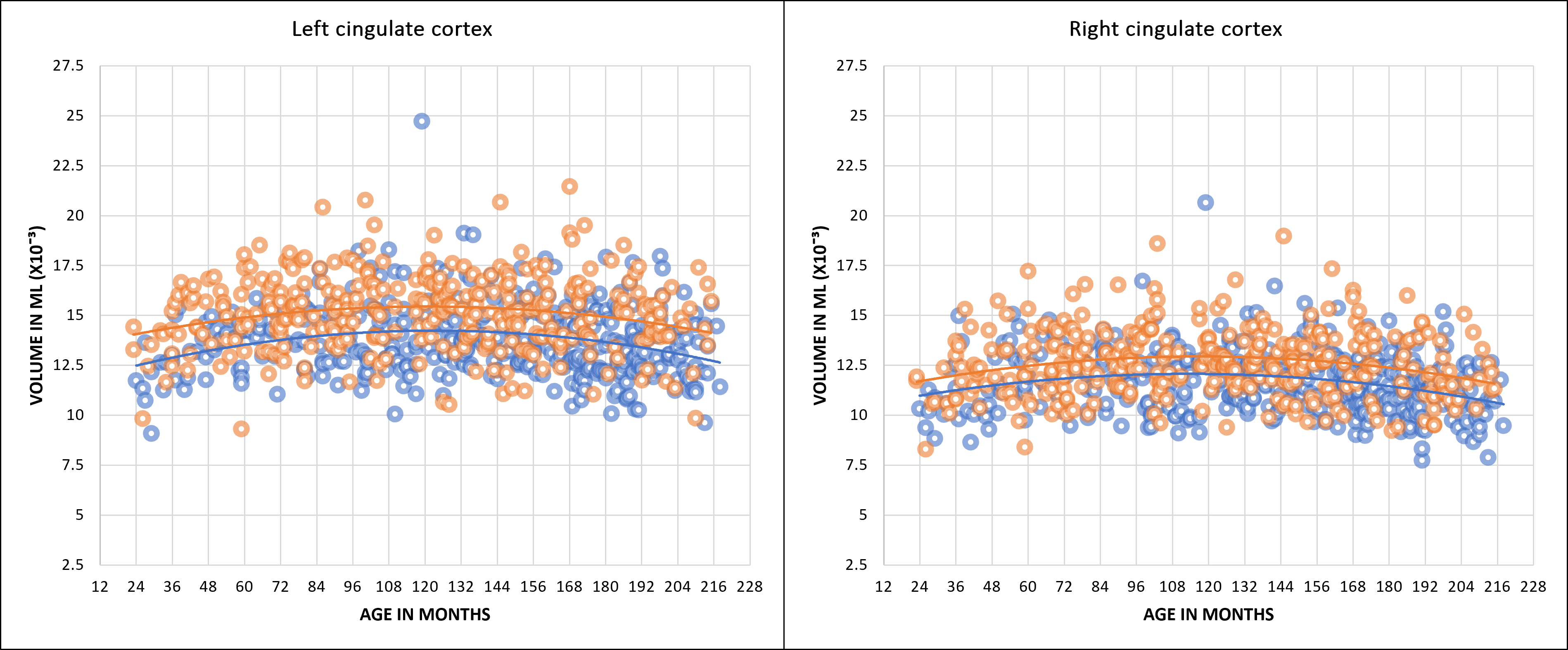

Supplement: Supplementary file 1 [file children-10-00477-s001.zip › children-2244551-supplementary/Figure S6.tif]

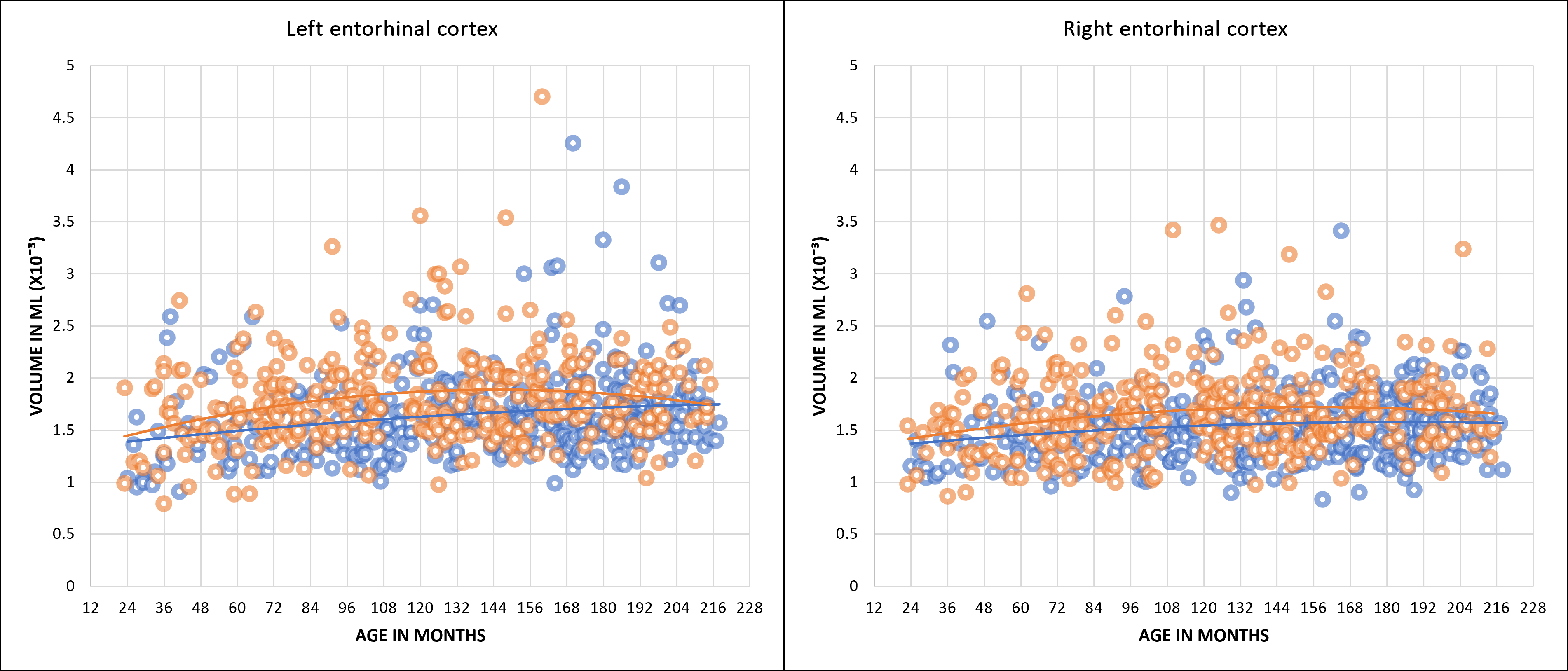

Supplement: Supplementary file 1 [file children-10-00477-s001.zip › children-2244551-supplementary/Figure S7.tif]

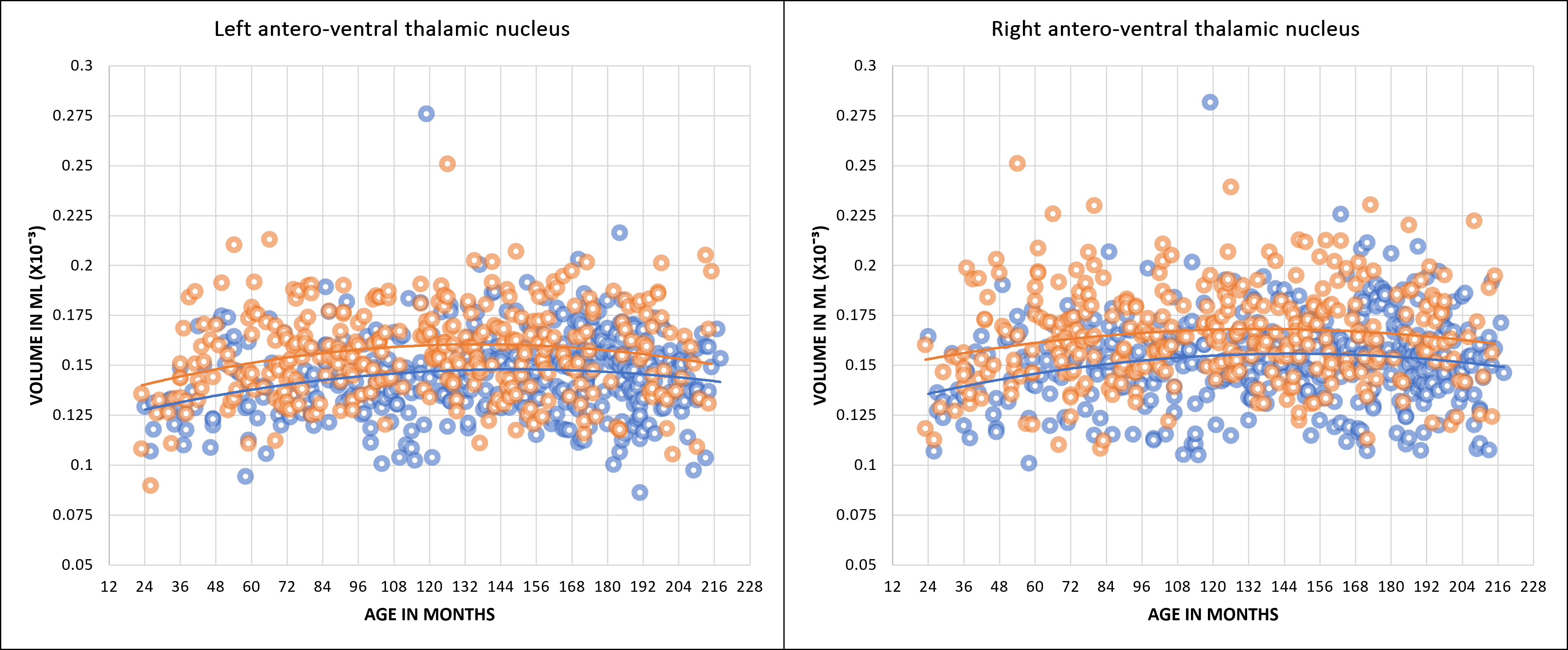

Supplement: Supplementary file 1 [file children-10-00477-s001.zip › children-2244551-supplementary/Figure S8.tif]

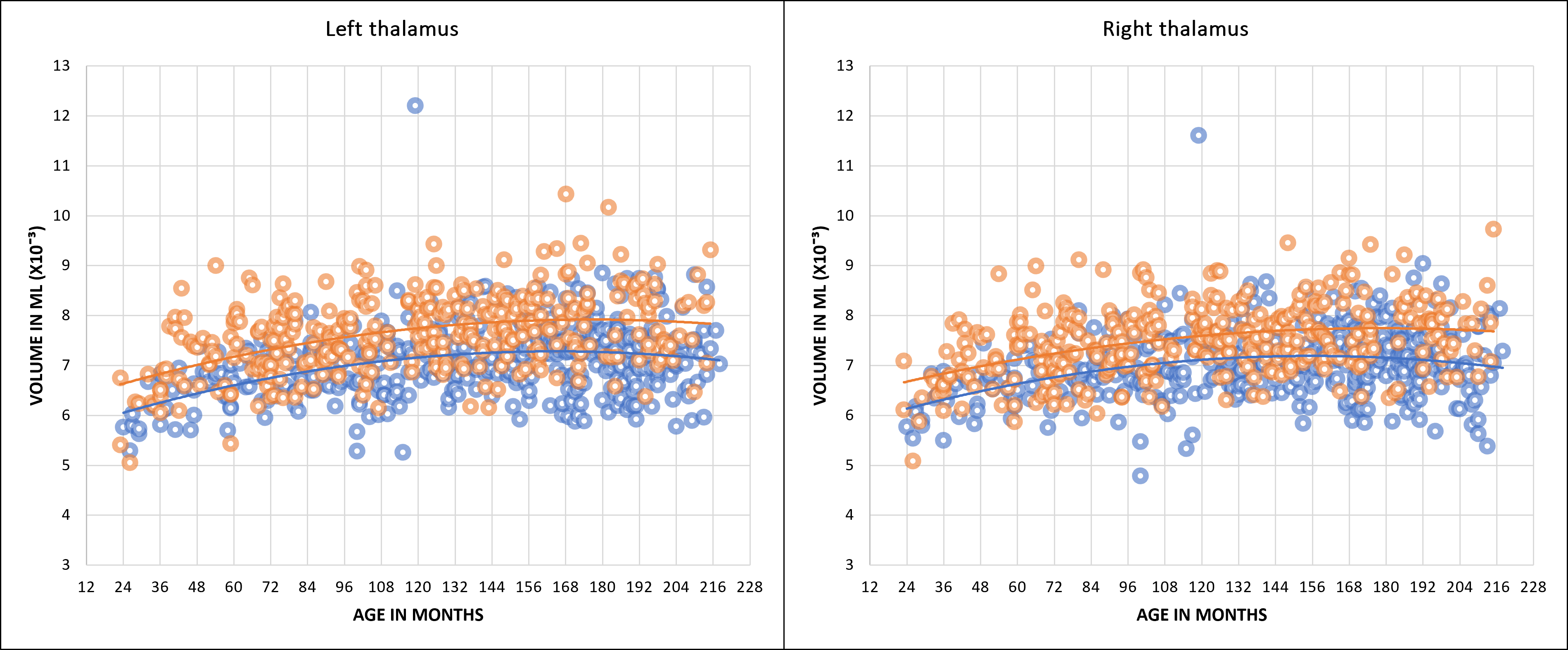

Supplement: Supplementary file 1 [file children-10-00477-s001.zip › children-2244551-supplementary/Figure S9.tif]
